# Pneumonia Caused by Three Separate Microorganisms Simultaneously in a Patient Infected with Human Immunodeficiency Virus

**DOI:** 10.7759/cureus.7804

**Published:** 2020-04-23

**Authors:** Sohaip Kabashneh, Samer Alkassis, Hammad Ali

**Affiliations:** 1 Internal Medicine, Wayne State University, Detroit Medical Center, Detroit, USA

**Keywords:** community-acquired pneumonia, hiv, pneumococcal bacteremia, bronchoscopy, bal

## Abstract

Community-acquired pneumonia (CAP) is usually caused by a single microorganism. *Streptococcus pneumoniae* is the most common organism associated with CAP. However, in immunocompromised patients, especially those infected with human immunodeficiency virus (HIV), pneumonia may be caused by multiple organisms simultaneously. This report describes a previously healthy 29-year-old man who presented with acute CAP. Blood tests showed that he was positive for HIV antigen/antibody, and urinalysis showed that he was initially positive for pneumococcal antigen. Although blood cultures showed growth of *Streptococcus pneumoniae*, he did not respond to invasive anti-pneumococcal treatment with ceftriaxone and vancomycin. Rather, his pneumonia worsened, and he was intubated for hypoxic respiratory failure. His bronchoalveolar lavage fluid was positive for *Pneumocystis pneumonia* and methicillin-resistant *Staphylococcus aureus*. These findings indicate that pneumonia in immunocompromised patients may be caused by multiple organisms. Patients who fail to respond to treatment for a single identified organism should be suspected of being infected with other pathogenic organisms.

## Introduction

The incidence of community-acquired pneumonia (CAP) among adults in the United States is 16 to 23 per 1000 persons per year, with approximately 30% of these patients requiring hospitalization [1,2]. *Streptococcus pneumoniae* is the most commonly detected bacterial species in patients with CAP [3,4]. Rates of bacterial pneumonia are 10 times higher in patients infected with human immunodeficiency virus (HIV) than in healthy individuals, despite the former receiving antiretroviral therapy. Bacterial pneumonia is currently the most frequent cause of overall and pulmonary infections in HIV-infected patients, as well as the most frequent diagnosis at hospital admission [5-7].

Pneumonia in HIV-infected patients is frequently caused by simultaneous infection with multiple organisms, making pneumonia management very challenging in these patients. Opportunistic infections in HIV-infected patients can include bacterial species in 60% of cases, mycobacteria in 18%, viruses in 5%, and fungi, including *Pneumocystis pneumonia* in 20% [7]. Infections with all of these microorganisms must be considered to optimize treatment. 

## Case presentation

**Figure 1:**
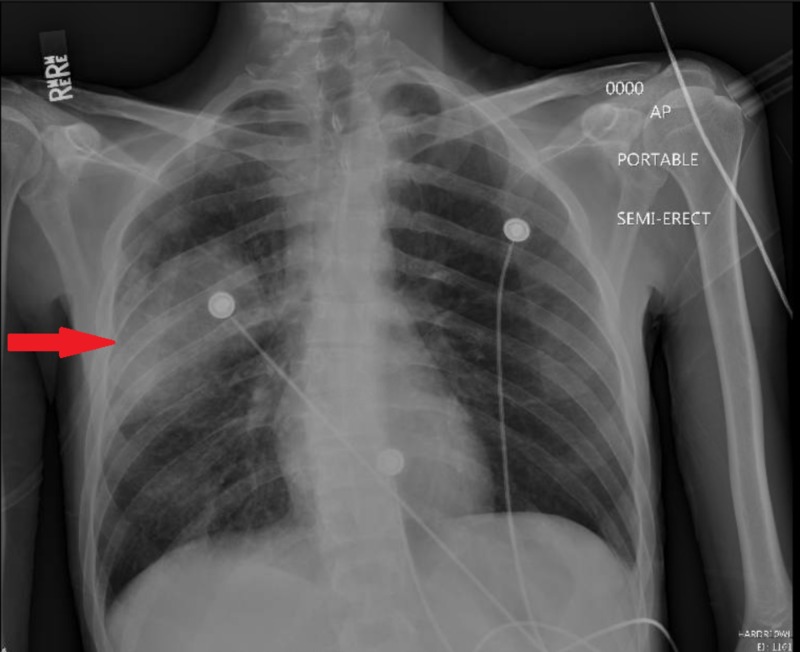
Chest X ray on admission demonstrating a right middle lobe infiltrate (red arrow).

A 29-year-old man with a previous history of alcohol use disorder and injection drug use presented to our hospital with fever, cough, and shortness of breath, along with chronic diarrhea lasting for a few months. The patient reported feeling ill for two days prior to presentation but subsequently felt feverish and sweaty, prompting him to visit the emergency department. On examination, the patient was febrile with a temperature of 39.1 °C (102.3 °F) and tachycardic with a heart rate of 101 beats per minute. His blood pressure was borderline, 95/60 mmHg; he was hypoxic with 89% breathing in room air, and he was in a moderate degree of distress. Examination of his lungs revealed decreased air entry on the right side with some crackles, whereas examination of his mouth showed oral thrush. The findings of his abdominal examination were unremarkable, with no evidence of organomegaly or tenderness to abdominal palpation. Biochemical and hematological investigations revealed a healthy leukocyte count of 7,500 cells/µL (neutrophils 94%), low hemoglobin of 10.4 gm/dL (healthy mean corpuscular volume of 99), a healthy creatinine level of 0.4 mg/dL, an elevated aspartate transaminase level of 216 U/L (reference range is <39 U/L), a healthy alanine transaminase level of 49 U/L (reference range is <52 U/L), and an elevated total bilirubin level of 1.5 mg/dL (reference range is <1.00 mg/dL). A chest x-ray showed focal consolidation in the right middle lobe (Figure 1) . Blood cultures ,* S. pneumoniae* urine antigen and *Legionella pneumophila *urine antigen were performed, and the patient was started on empirical treatment with ceftriaxone and azithromycin. Further assessment of the patient revealed that he was in a monogamous relationship with a male partner and regularly participated in unprotected anal intercourse. A fourth-generation rapid HIV antigen-antibody test was, therefore, performed.

**Figure 2:**
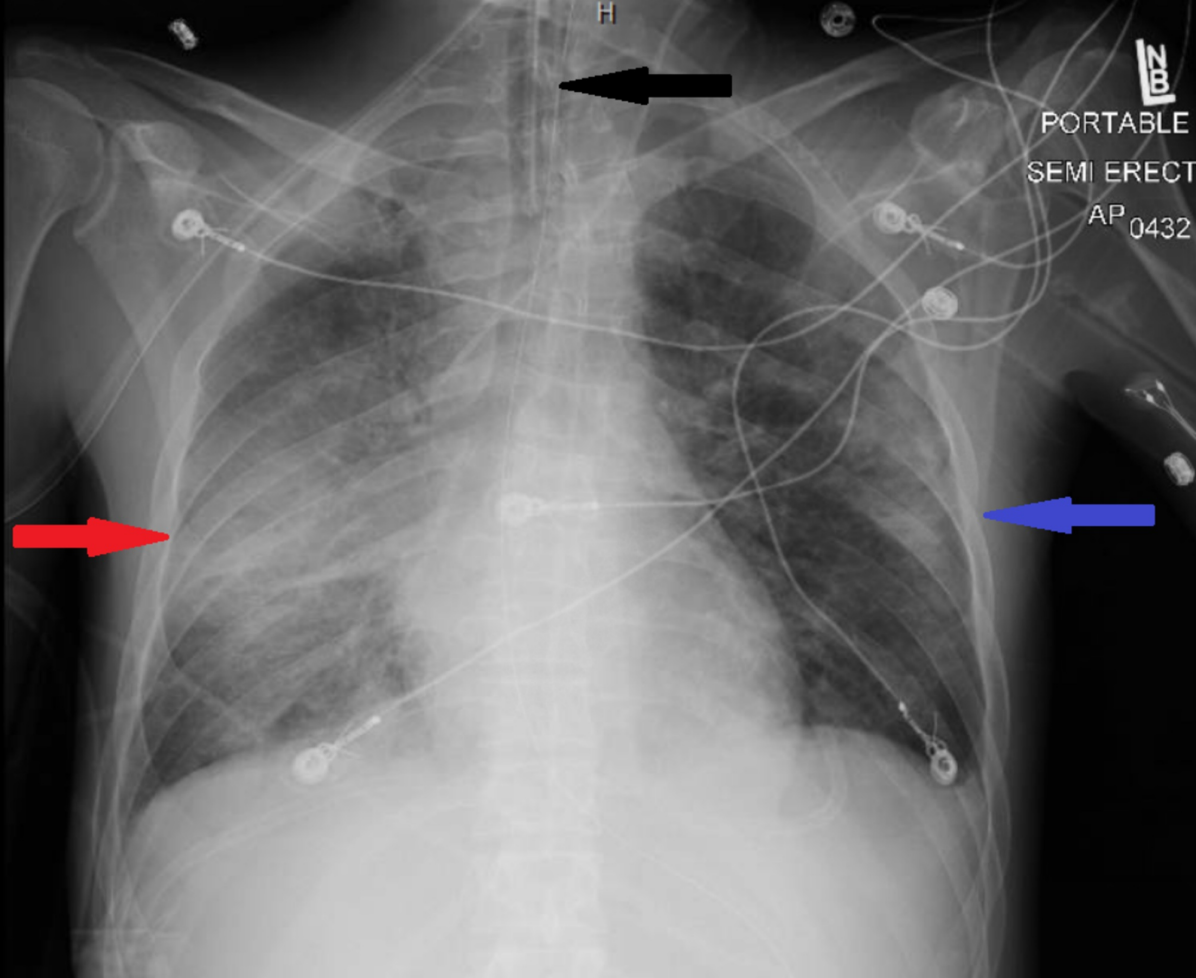
Chest x ray on day 3 demonstrating a worsening of the infiltrate on right side (red arrow) along with the appearance of a new infiltrate on the left side (blue arrow) and Interval placement of endotracheal tube (black arrow).

**Figure 3:**
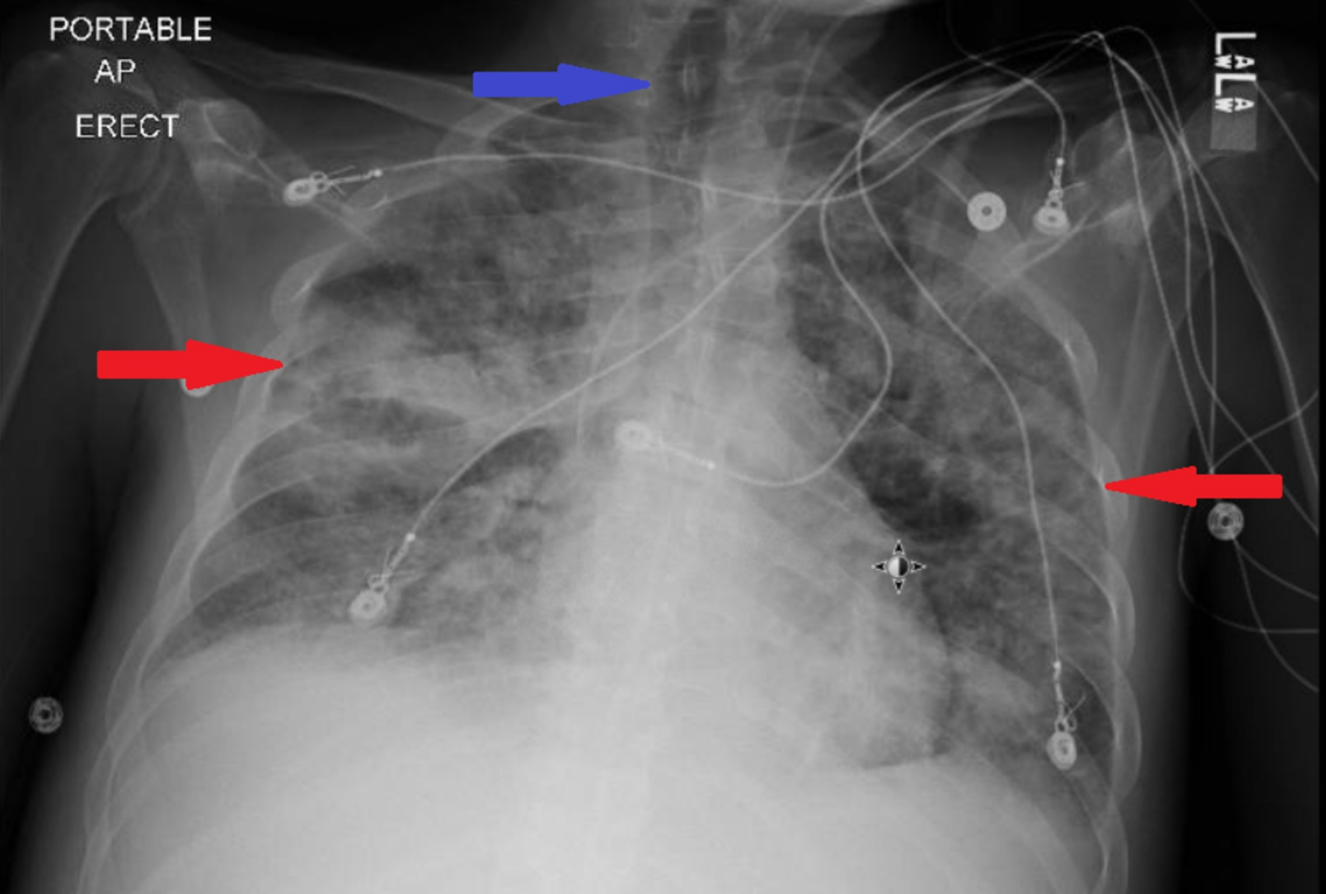
Chest x ray on day 5 demonstrating a bilateral infiltrate (red arrows), radiographic changes can lag behind clinical improvement. Endotrachial tube is no longer visible (blue arrow) .

**Figure 4:**
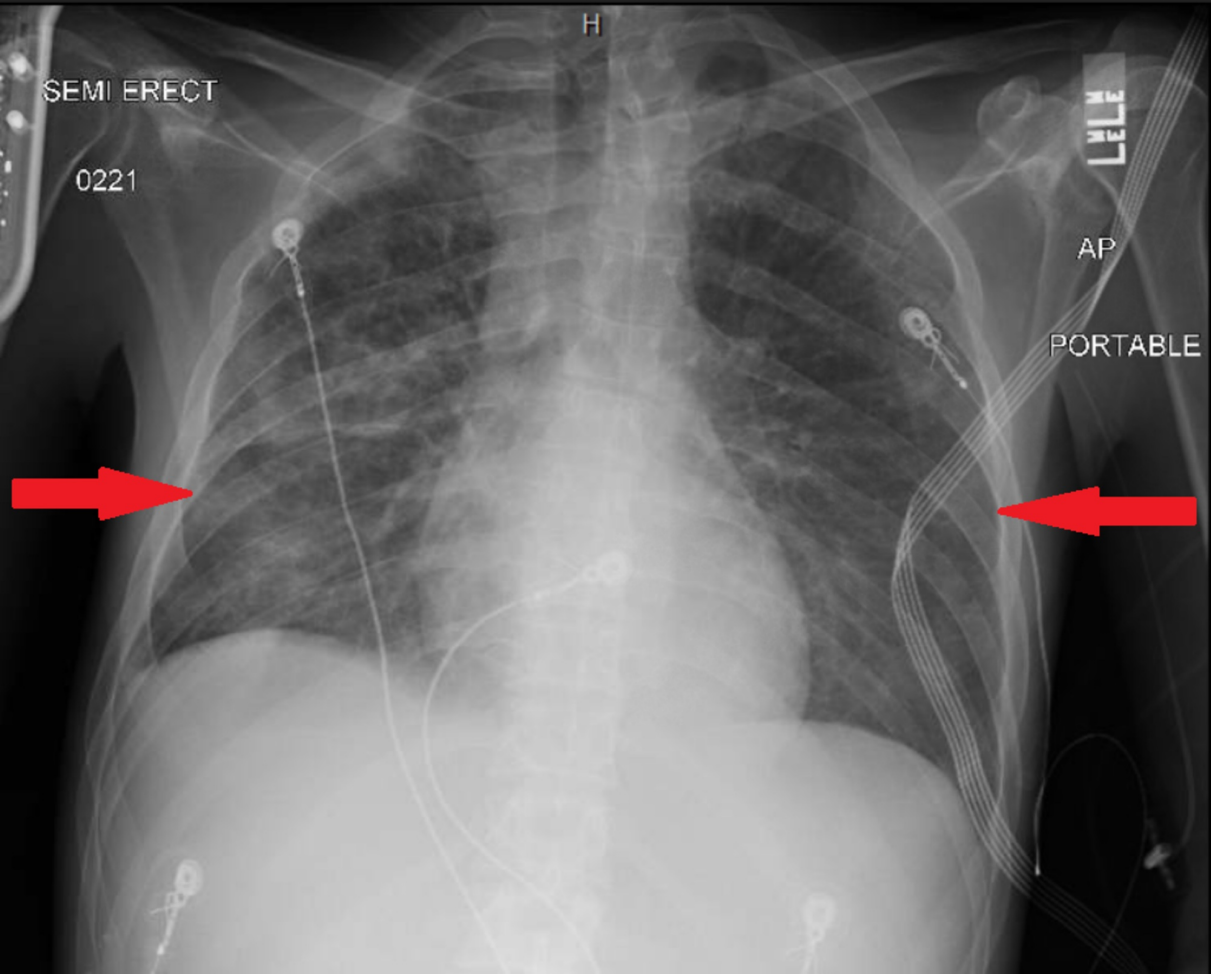
Chest x ray on day twelve , 2 days prior to discharge , demonstrating significant improvement in the previously noted bilateral infiltrates (red arrows).

On the second day of admission, the patient continued to experience febrile episodes, was still coughing and experiencing shortness of breath, and was still hypoxic. Results of urinalysis showed that he was positive for *S. pneumoniae* antigen, and rapid HIV testing was also positive with a CD4 count of 18 cells/mm3. He was preliminary diagnosed with pneumonia due to *S. pneumoniae*. Treatment with ceftriaxone was therefore continued, but all other antibiotics were discontinued. On the third day, however, his condition deteriorated, and he developed respiratory failure. The patient was intubated with ventilator settings that included a respiratory rate of 18 breaths/minute, a tidal volume of 460 mL, a fraction of inspired oxygen of 60%, and a positive end-expiratory pressure of 5 cm H_2_O. His blood culture was positive for pan-susceptible S. *pneumoniae*, and repeated chest x-rays showed bilateral infiltrates (Figure 2). Military tuberculosis was ruled out as three sputum samples were negative. Vancomycin was added, and bronchoscopy was performed to determine the presence of opportunistic infections. His bronchoalveolar lavage fluid was positive for *Pneumocystis jirovecii* and methicillin-resistant *Staphylococcus aureus* . He was therefore started on trimethoprim-sulfamethoxazole and corticosteroids. Two days later, his condition improved, and he was extubated to a high-flow nasal cannula (Figure 3). He continued to improve and was discharged after being hospitalized for two weeks (Figure 4).

## Discussion

This report describes a young homosexual man with a history of injection drug use and unprotected intercourse who presented with community acquired pneumonia due to *S. pneumoniae*. However, the lack of response to antibiotics and newly diagnosed AIDS required further investigation, which revealed concurrent pulmonary infection with *Pneumocystis jirovecii* and methicillin-resistant *Staphylococcus aureus*.

Bacterial pulmonary infections occur frequently in persons infected with HIV. The development of highly active antiretroviral therapy has led to advances have in the care and treatment of patients with HIV, altering the presentation of HIV-associated pulmonary diseases. Pneumonia caused by bacterial infections, particularly *S. pneumoniae*, remain commonplace, whereas opportunistic infections with agents such as *P. jirovecii* remain a concern in patients without adequate access to optimal medical care [8]. The annual incidence of bacterial pneumonia in HIV-seropositive patients ranges from 5.5 to 29 per 100, compared with 0.7 to 10 per 100 in HIV-seronegative patients [9-11].

Although bacterial pneumonia can occur throughout the course of HIV infection, it is more frequent in individuals with advanced immunosuppression [10,12]. Moreover, the incidence of bacterial pneumonia was shown to directly correlate with CD4 count [11]. Other traditional risk factors that may be associated with pneumonia include pre-existing lung disease (e.g., bronchiectasis or chronic obstructive pulmonary disease), heavy alcohol use, injection drug use, neutropenia, and severe malnutrition. Bacterial pneumonia in HIV-infected patients is associated with a permanent decline in pulmonary function [13] and a two-fold to five-fold increase in long-term mortality compared with CD4-matched controls [11,13,14].

Respiratory symptoms and suspected pneumonia are evaluated in HIV infected individuals to establish a definitive diagnosis, thereby allowing the initiation of appropriate treatment. However, definitive diagnoses may require invasive diagnostic procedures such as bronchoscopy, as well as sophisticated laboratory techniques. The clinical and radiographic presentations of HIV-associated opportunistic pneumonias overlap, and HIV infected individuals may present concurrently with pneumonia caused by more than one agent. Bacterial pneumonia may be the first manifestation of underlying HIV infection. Thus, HIV infection should be suspected in any patient presenting with bacterial pneumonia, especially in patients with no other risk factors for pneumonia and in patients with recurrent pneumonia. Similar to findings in non-HIV infected individuals, *S. pneumoniae* and *Haemophilus* species are the most frequently identified causes of CAP. *Pseudomonas aeruginosa* and *Staphylococcus aureus* are more frequent causes of CAP in patients with than without HIV infection [15]. The clinical and radiological presentations of lower respiratory tract infections in HIV-infected patients are quite variable. Clinical presentations are more severe, and radiological imaging more atypical in severely than in mildly immunosuppressed HIV infected individuals [16].

Chest radiography is the first step in the diagnostic evaluation of persons with suspected pneumonia. Specific findings on chest radiography, along with CD4 cell count, often suggest a differential diagnosis and plan for management and treatment. Selected laboratory tests may be indicated to assess for specific diseases or disease severity (e.g., arterial blood gas). In some cases, computed tomography of the chest may be indicated. Where feasible, further evaluation should seek to establish a definitive diagnosis, with microbiologic evaluation of sputum, including via staining and culturing, as well as bronchoscopy in selected patients [15].

A suboptimal response to empiric therapy or to the treatment of an identified pathogen in patients with HIV-related pulmonary diseases suggests the need for a definitive or additional diagnosis. It is also essential to exclude pulmonary tuberculosis in HIV-seropositive patients with CAP who fail to respond appropriately to initial antibiotic therapy, even if another etiological pathogen has been identified [15].

## Conclusions

This report describes a young man who presented with what seemed like a straightforward CAP due to* S. pneumoniae*. He was found to be infected with HIV and to have a low CD4 count of 18 cells/mm3 but failed to respond to appropriate CAP treatment. The lack of improvement may have been due to complications of pneumonia, including abscess and acute respiratory distress syndrome, or to simultaneous infection with multiple organisms. These findings suggest that simultaneous infection with multiple organisms may be responsible for CAP in HIV-infected patients.
